# Shutting off the fuel supply to target metabolic vulnerabilities in multiple myeloma

**DOI:** 10.3389/fonc.2023.1141851

**Published:** 2023-06-08

**Authors:** Priyanka S. Rana, Krishna Goparaju, James J. Driscoll

**Affiliations:** ^1^ Division of Hematology and Oncology, Department of Medicine, Case Western Reserve University, Cleveland, OH, United States; ^2^ Immune Oncology Program, Case Comprehensive Cancer Center, Cleveland, OH, United States; ^3^ Adult Hematologic Malignancies & Stem Cell Transplant Section, Seidman Cancer Center, University Hospitals Cleveland Medical Center, Cleveland, OH, United States

**Keywords:** metabolism, multiple myeloma, proteasome inhibitor, oxidative phosphorylation, glycolysis, fatty acid synthesis

## Abstract

Pathways that govern cellular bioenergetics are deregulated in tumor cells and represent a hallmark of cancer. Tumor cells have the capacity to reprogram pathways that control nutrient acquisition, anabolism and catabolism to enhance their growth and survival. Tumorigenesis requires the autonomous reprogramming of key metabolic pathways that obtain, generate and produce metabolites from a nutrient-deprived tumor microenvironment to meet the increased bioenergetic demands of cancer cells. Intra- and extracellular factors also have a profound effect on gene expression to drive metabolic pathway reprogramming in not only cancer cells but also surrounding cell types that contribute to anti-tumor immunity. Despite a vast amount of genetic and histologic heterogeneity within and between cancer types, a finite set of pathways are commonly deregulated to support anabolism, catabolism and redox balance. Multiple myeloma (MM) is the second most common hematologic malignancy in adults and remains incurable in the vast majority of patients. Genetic events and the hypoxic bone marrow milieu deregulate glycolysis, glutaminolysis and fatty acid synthesis in MM cells to promote their proliferation, survival, metastasis, drug resistance and evasion of immunosurveillance. Here, we discuss mechanisms that disrupt metabolic pathways in MM cells to support the development of therapeutic resistance and thwart the effects of anti-myeloma immunity. A better understanding of the events that reprogram metabolism in myeloma and immune cells may reveal unforeseen vulnerabilities and advance the rational design of drug cocktails that improve patient survival.

## 1 Introduction

The hallmarks of cancer constitute an organizing principle to rationalize the complexities of neoplastic disease (1–4). Six biological capabilities - sustaining proliferative signaling, evading growth suppressors, resisting cell death, enabling replicative immortality, inducing angiogenesis, and activating invasion and metastasis acquired during the multistep development of human tumors - were initially identified as the hallmarks of human cancers (1). Genomic instability underlies these features that promote genetic diversity and intratumoral heterogeneity. Recently, reprogramming of energy metabolism and evading immune destruction have also been recognized as cancer hallmarks (2–4). Cancer cells sustain prodigious anabolic requirements that exceed those of neighboring somatic cells. Metabolic pathways in cancer cells are reprogrammed to achieve the required bioenergetic, biosynthetic and redox demands. Reprogramming of energy metabolism is also required to support continuous cell growth and proliferation, replacing the metabolic program that operates in most healthy tissues and fuels physiological operations within cancer cells (4–6). Tumorigenesis stems from the direct and indirect consequences of oncogenic mutations to reprogram key metabolic pathways (4, 7–12). Cancer-associated metabolic reprogramming also alters the level of key intracellular and extracellular metabolites (2, 11, 12).

Tumors display an added dimension of complexity since they contain a repertoire of recruited, ostensibly normal cells that contribute to the acquisition of hallmark traits by creating the tumor microenvironment (TME) (7–15). The TME is comprised of heterogeneous and interactive cell types including cancer cells and cancer stem cells surrounded by a multitude of recruited stromal cell and immune cell types. Cellular metabolism is reprogrammed in cancer cells by tumor-intrinsic and extrinsic factors. Cancer cells proliferate within the tumor permissive bone marrow (BM) and are surrounded by a complex environment that consists of cellular and acellular components, e.g., blood and lymph vessels, fibroblasts, endothelial cells, numerous immune cell types, osteoblasts, osteoclasts, pericytes, platelets, hematopoietic stem cells and other cell types. In addition, cancer cells are also influenced by surrounding cytokines, extracellular vesicles, cartilage, fat, bone and the extracellular matrix these reside within the BM milieu (10–15).

Oncometabolites are metabolites that aberrantly accumulate from distorted metabolism and are considered novel pathognomonic hallmarks in certain human cancers, e.g., glioma, leukemia, neuroendocrine tumors, and renal cancer (16–19). Oncometabolites have been shown to play a pivotal role in neoplastic transformation, cancer metabolism, and the development of therapeutic resistance. As a consequence of gain-of-function mutations and loss of tumor suppressors, oncometabolites accumulate within cancer cells and within the TME. For example, mutations in isocitrate dehydrogenase 1 and 2 (*IDH1/2*) occur in a subset of acute myeloid leukemia (AML) patients and *IDH2* mutant leukemic cells produce elevated levels of the oncometabolite d-2-hydroxyglutarate (D2-HG) (17, 18). D2-HG is a structural homolog and antagonist of the Krebs cycle intermediate α-ketoglutarate (α-KG) that disrupts the Krebs cycle leading to metabolic and epigenetic derangements. D2-HG changes the catalytic activity of α-ketoglutarate–dependent dioxygenases leading to genome-wide histone and DNA methylation alterations (16–19).

## 2 Linking altered cellular metabolism to multiple myeloma

Multiple myeloma (MM) is a cancer of terminally-differentiated plasma cells (PCs) that accumulate and proliferate predominantly within the tumor permissive BM microenvironment (20–25). PCs are primary effectors of humoral immunity and function as antibody-producing factories that secrete vast amount of immunoglobulins. PC proliferation within the BM leads to increased production and circulation of the monoclonal (M-spike) protein in serum and/or urine (6, 26). Cardinal clinical features of MM include anemia, hypercalcemia, renal impairment and myeloma-related bone lesions (6, 20–22). The clinical course of nearly all MM patients is characterized by cycles of continuously shortening periods of remission followed by relapse. The prevalence of obesity, cardiovascular disease and diabetes increases with age and elderly patients diagnosed with MM generally present with these concurrent co-morbidities (6, 27–32). The prognosis of MM patients has significantly improved over the past two decades, primarily due to the incorporation of novel agents developed based upon the biology of disease (20, 22, 33). MM cells synthesize and secrete vast amounts of protein, especially immunoglobulins, and have adapted to withstand an enhanced capacity for unfolded polypeptides. Hence, MM cells are exquisitely sensitive to drugs that disrupt protein homeostasis, e.g., proteasome inhibitors (PIs). Although PIs represent a highly effective anti-myeloma therapy and transformed the management of MM, drug resistance inevitably emerges through compensatory protein clearance mechanisms, e.g., the aggresome+autophagy pathway (34). Genome-wide profiling identified individual microRNAs (miRs), e.g., miR-29b, that were differentially expressed in bortezomib-resistant MM cells compared to drug-naive cells. The highly distinct function and specialized habitat of MM cells shapes the circuitry of intracellular pathways that contribute to drug resistance (35).

Genomic, proteomic and metabolic changes in myeloma cells stimulates their clonal evolution and expansion that eventually leads to the emergence of drug resistant clones that are responsible for disease relapse (36–38). Altered cellular metabolism also reduces the anti-myeloma effect of standard-of-care agents, e.g., PIs and immunomodulatory drugs (IMiDs). Metabolic changes within the TME further decreases the beneficial anti-myeloma effects of PIs and IMiDs, monoclonal antibodies and cellular immunotherapies (14, 15, 23, 36). Despite the development of novel anti-myeloma drugs over the past two decades, disease heterogeneity, high-risk disease, early relapse and treatment resistance remain challenges (14, 20, 22, 24, 33). Moreover, subclonal heterogeneity of PCs evolves alongside disease progression through the selection of genetically and metabolically adapted subclones (37, 38). Importantly, the incidence of MM is associated with metabolic syndrome and inflammatory cytokines, while the anti-diabetic agent metformin that lowers blood glucose levels and statins, which lower the level of low-density lipoprotein (LDL) cholesterol, are positive prognostic factors in patients diagnosed with MM (39–42).

## 3 Metabolic pathways altered in multiple myeloma

MM cells employ specialized metabolic programs that differ from neighboring, untransformed somatic cells to sustain their extraordinary anabolic and catabolic needs (6, 42–45). Features of altered metabolism in MM include deregulated uptake and metabolism of glucose and amino acids especially glutamine, capacity to acquire scarce nutrients, enhanced glycolytic and tricarboxylic acid (TCA) cycle intermediates, elevated nicotinamide adenine dinucleotide phosphate (NADPH) production and elevated level of fatty acid (FA) synthesis.

### 3.1 Glucose metabolism

The glycolytic enzyme hexokinase II (*HKII*) is overexpressed in MM cells relative to PCs from healthy donors (46). Ikeda et al. found that hypoxia-inducible HKII impaired glycolysis and contributed to autophagy activation as well as the acquisition of an anti-apoptotic phenotype in myeloma cells. To detect candidate genes crucial for the acquisition of hypoxia-inducible autophagy, a comprehensive expression analysis was performed using MM patient samples incubated under normoxia or hypoxia. Hypoxic stress upregulated glycolytic genes (*PFKFB4*, *ENO2*, *ALDOC*, *PFKFB3*, *HK2*, *PFKP, GPI, PGK1*, *LDHA*, *ALDOA*, *ENO1*, *PKM*, and *GAPDH*) including *HKII* in samples obtained from MM patients (46). These results suggest that hypoxia-drive event may permit myeloma cells to metabolize glucose in an energetically favorable multi-step process. Antisense oligonucleotide (ASO) directed against *HKII (HII-ASO1*) suppressed *HKII* expression in MM cell cultures and in MM patient tumor cells xenografted into murine models (47). *HKII-ASO1* shows selective *HKII* inhibition to support the clinical development of this approach. Aerobic glycolysis also activates the TCA cycle to produce NADPH and glutathione (GSH) which reduces oxidative stress. Since oxidative damage is essential for bortezomib-mediated cytotoxicity, drug resistance may be accompanied by increased tolerance towards oxidative insults. Soriano et al. showed that PI-adapted myeloma cells tolerate subtotal proteasome inhibition owing to metabolic adaptations that favor the generation of NADPH reducing equivalents, supported by oxidative glycolysis (48).

Lactate dehydrogenase A (*LDHA*) expression is increased in relapsed MM patients to suggest that glucose metabolism is enhanced (49). Proliferator-activator receptor-γ coactivator-1β (*PGC-1β)* and *LDHA* are highly expressed in MM cells and *LDHA* is upregulated by *PGC-1β* through the *PGC-1β/RXRβ* axis by acting on the *LDHA* promoter. Overexpression of *PGC-1β* or *LDHA* potentiated glycolysis metabolism and increased cell proliferation and tumor growth. Conversely, knockdown of either *PGC-1β* or *LDHA* suppressed glycolysis, increased reactive oxygen species (ROS) formation and apoptosis, suppressed tumor growth and enhanced mouse survival. Liu et al. investigated whether excess glucose induced hypoxia-inducible factor-1α (HIF-1α) and stimulated glucose metabolism and cell migration in pancreatic cancer cells (50). The authors studied wild-type (WT) MiaPaCa2 pancreatic cancer cells and a MiaPaCa2 subline transfected with an HIF-1α-specific small interfering (siRNA). Excess glucose stimulated the migration of WT and siRNA-treated MiaPaCa2 cells grown under normoxia and hypoxia, while glucose stimulated cell migration independent of HIF-1α. These studies indicated that excess glucose increases HIF-1α and ATP in hypoxic WT-MiaPaCa2 cells. Extracellular glucose levels and hypoxia regulate glucose metabolism independent of HIF-1α while glucose stimulates cell migration through HIF-1α-dependent and independent mechanisms.

The Warburg effect describes an increase in the rate of glucose uptake and preferential production of lactate, even in the presence of oxygen (51–53). Further evidence that Warburg’s

experiments on tumor tissue *in vitro* were valid *in vivo* was demonstrated in experiments on surviving tumor tissue and replicated in tumor-bearing animals (54, 55). The effect is clinically utilized in ^18^F-fluorodeoxyglucose (^18^F-FDG) positron emission tomography (PET) scans as sensitive diagnostic and prognostic tools (56, 57). Glucose is transported across the cell membrane through glucose transporters (GLUTs) through a facilitated diffusion mechanism (58–61). Owing to its elevated glycolytic gene profile, MM cells have been shown to be dependent on glycolysis and, therefore, susceptible to glycolysis inhibitors, e.g., GLUT inhibitors (58). Of the 14 GLUT subtypes, *GLUT1* overexpression is most associated with poor clinical outcomes in cancer cell lines and cancer patients (44, 47, 58, 61). In MM cells, *GLUT1* upregulation increases glucose uptake and enhances susceptible to GLUT1 inhibitors (61). MM cells are also dependent on *GLUT4* for glucose uptake, survival, and elevated expression of the anti-apoptotic protein *Mcl-1*, that has been associated with tumorigenesis, poor prognosis, and drug resistance (58).

Upregulation of the GLUT membrane transporters, e.g., GLUT1 GLUT4, GLUT8 and GLUT11, increases the level of glycolytic metabolites in MM cells. The Federal Drug Administration (FDA)-approved HIV protease inhibitor ritonavir demonstrates an off-target inhibitory effect on GLUT4 as well as a dose-dependent inhibitory effect on glucose uptake and proliferation in L363 and KMS11 cells (62). However, a subset of MM cells survive glucose deprivation or ritonavir treatment, possibly through mitochondrial oxidative phosphorylation (OXPHOS). Targeting the mitochondrial complex I using the FDA-approved anti-diabetes drug metformin combined with ritonavir induced apoptosis in primary MM cells. The PI3K/AKT pathway, through mTOR-dependent activity, is linked to increased glucose metabolism and may explain the elevated levels of glycolytic intermediates seen in MM cells (63–66). The combination also suppressed AKT and mTORC1 phosphorylation and downregulated *Mcl-1* expression (62).

LDH, which converts pyruvate and NADH to lactate and NAD^+^, is elevated in ~10% of patients with newly-diagnosed, symptomatic MM (67). *HIF-1α* is upregulated in drug resistant MM cells and leads to enhanced lactate production and the accumulation of glycolytic metabolites (68). *HIF-1α* upregulation is associated with metastasis, unfavorable prognosis, and reduced OS in cancer patients (68, 69). Since bortezomib decreases HKII activity in MM cells grown under hypoxic conditions and loss of HKII decreases LDHA activity, targeting LDHA could enhance effects of bortezomib (45). Indeed, inhibition of *HIF-1α* and *LDHA* have been shown to restore sensitivity to bortezomib and melphalan in MM cells (45). FX11 is a selective and potent LDHA inhibitor which reduces ATP levels by inducing oxidative stress and ROS production (70) (**Table 1**). PX-478 selectively inhibits HIF-1α to suppress cell migration, angiogenesis and drug resistance (71). Pyruvate kinase M2 (PKM2) regulates glycolysis and promotes tumor cell survival and proliferation (86). Never in mitosis gene A (NIMA)-related kinase 2 [*NEK2*] increases the PKM2/PKM1 ratio by splicing PKM to promote enhanced glycolysis that drives oncogenesis (54).

**Table 1:** Pharmacologically targeting metabolic vulnerabilities in hematologic malignancies.

Metabolic Pathway	Target	Drug	Mechanism of Action
**Glycolysis**	HIF-1α	PX-478 (Phase I, NCT00522652)	Decreases nuclear HIF-1α protein levels to reduce HIF-1α (71).
	LDHA and HK2	FX11 (Preclinical)	Inhibits aerobic glycolysis (70).
	GLUT4	Ritonavir (Phase I, NCT02948283)	Cytostatic and/or cytotoxic effects by chemosensitizing tumor cells both *in vitro* and *in vivo* (62, 72).
	GLUT1	Vincristine (Phase II, NCT00003493)	Inhibits microtubule formation in mitotic spindle, resulting in an arrest of dividing cells at the metaphase stage (73).
		Bortezomib (Phase IV, NCT00257114)	Binds reversibly to the chymotrypsin-like subunit of the 26S proteasome, resulting in its inhibition and preventing the degradation of various pro-apoptotic factors (48).
		WZb117 (Preclinical)	Inhibits passive sugar transport in human erythrocytes and cancer cell lines and, by limiting glycolysis (74).
		Phloretin (Preclinical)	Blocks cyclins and cyclin-dependent kinases and activates mitochondria-mediated cell death to promote cell death (61, 75).
	Hexokinase	Vincristine	See above.
		Bortezomib	See above.
**OXPHOS**	Glycerophosphate dehydrogenase	Metformin (Phase II, NCT04850846)	Inhibits MM proliferation by inducing cell cycle arrest and apoptosis (39, 40, 76).
**Amino acid metabolism**	Glutaminase	Benzophenanthridinone 968 (Preclinical)	Promotes apoptosis in both human MMCL and patient primary cells (77, 78).
		CB-839 (Telaglenastat) (Phase I, NCT03798678)	Allosteric, noncompetitive inhibitor of both splice variants of the broadly expressed glutaminase-1. Enhanced CFZ-induced ER stress and apoptosis, characterized by a robust induction of ATF4 and CHOP and the activation of caspases (79).
	Guanine and Guanosine GSH	Melphalan (Phase II, NCT02669615)	Alkylates guanine and causes linkages between strands of DNA leading to cytotoxicity in dividing and non-dividing cells (80).
	SNAT1	α-Methylamino-isobutyric acid (Preclinical)	Competitive inhibitor of the neutral amino acid transport A system which decreases glutamine uptake and reduces cell growth (81, 82).
	ASCT2	V-9302 (Preclinical)	Blocks ASCT2 to attenuate cancer cell growth and proliferation, increase cell death, increase oxidative stress, to contribute to anti-tumor responses *in vitro* and in murine models *in vivo* (83).
	LAT1	Nanvuranlat (JPH203) (Phase I, in solid tumors, PMID: 32198649)	Inhibits essential amino acids uptake in tumor cells to activate apoptosis (84).
**Fatty acid metabolism**	Carnitine palmitoyltransferase-1 (CPT1)	Etomoxir	Inhibits β-oxidation and *de novo* fatty acid synthesis in MM cells (43, 85).
	Fatty acid synthase (FASN)	Orlistat	Inhibits lipases and induces apoptosis in myeloma cells (43, 85).

The HK isoform HKII is the rate-limiting step in aerobic glycolysis and is overexpressed in many cancers including MM (87, 88). Vincristine and bortezomib suppressed GLUT-1 and HK expression to induce apoptosis in MM cells (73), while WZb117 and phloretin inhibited GLUT-1 activity to decrease glucose uptake with synergistic anti-tumor effects in leukemia, lung, colon and breast cancers (74, 89, 90). Under hypoxic conditions, phloretin enhanced the effects of daunorubicin and overcame hypoxia-conferred drug resistance (91). Targeting glucose consumption through enzymatic regulators and transporters could serve as an effective anti-myeloma therapy.

### 3.2 Amino acid metabolism

Glutamine is an abundant amino acid crucial for cell proliferation, differentiation, apoptosis, and cytokine production (92). Glutamine is needed in MM cells for nucleic acid biosynthesis, to generate energy in the TCA cycle and to support increased amino acid and FA synthesis. MM cells are particularly dependent on extracellular glutamine since they exhibit high glutaminase levels and low glutamine synthetase expression. Glutamine depletion prevents MM growth and enhances sensitivity to anti-myeloma agents (77, 79, 84, 93–95). The histidine/large neutral amino acid transporter LAT1 (SLC7A5) glutamine transporter is overexpressed in MM cells and is associated with reduced overall survival (OS) (84). MM cells primarily rely upon the alanine, serine, cysteine transporter 2 (ASCT2/SLC1A5) and glutamine transporters for glutamine uptake. Targeting glutamine transporters, specifically ASCT2 inhibitors combined with the PI carfilzomib induced proteotoxic stress and ROS generation (81, 83). The need for extracellular glutamine makes glutamine transporters interesting targets for MM therapy.

Glutamine serves as an important energy source for cancer cells and glutamine deficiency or the glutaminase inhibitor benzophenanthridinone 968 induces apoptosis in MM cells (13, 78, 82). Benzophenanthridinone 968 effectively inhibits glutaminase and this inhibition induces apoptosis in MM cell lines (MMCLs) and patient primary tumor cells. Elevated expression of the glutamine transporters SNAT1, ASCT2 and LAT1, makes these an attractive target for anti-myeloma therapy (6). The prognostic significance of LAT1 in MM was investigated by immunohistochemistry to monitor the expression of LAT1 and its functional subunit, 4Fc heavy chain (CD98), on tumor cells in 100 newly diagnosed MM (NDMM) patients (84). *LAT1* overexpression was associated with high proliferation and poor prognosis in NDMM patients. LAT1 may be a promising pathological marker to identify high-risk MM.

### 3.3 Fatty acid metabolism

A lipid profiling study uncovered large differences in lipid composition as well as amino acid and energy profiles from NDMM, relapsed and/or refractory (RRMM), monoclonal gammopathy of unknown significance (MGUS) and healthy controls (96). The metabolomic profile was quite different between that observed with samples from healthy controls compared to that of samples from patients with either MGUS, NDMM or RRMM. Significant alterations in amino acid, lipid, and energy metabolism were observed between the different patient groups. Eight metabolites, i.e., free carnitine, acetylcarnitine, glutamate, asymmetric dimethylarginine and phosphatidylcholine species, differed between MGUS and NDMM patients, supporting the notion that metabolic changes occur during myelomagenesis. A second lipidomics study revealed upregulation of ceramides and phosphatidylethanolamines (PEs) and downregulation of phosphatidylcholines, sphingomyelin and one species of PE in MM patients (97). Increased sphingomyelinase expression in primary patient samples was found and inhibition of sphingomyelinase by GW4869 further increased bortezomib and melphalan-mediated cell death (80). Treatment of MM cells with ixazomib led to the accumulation of lipids. Pre-treatment of MM cells with docosahexaenoic acid (DHA) or eicosapentaenoic acid (EHA) also increased the sensitivity to bortezomib by altering the GSH metabolic pathway (98). Tirado-Velez et al. tested the hypothesis that inhibition of β-oxidation and *de novo* FA synthesis would reduce cell proliferation in myeloma cells (85). The authors found that the RPMI-8226, NCI-H929 and U-266B1 cells displayed increased FA oxidation (FAO) and elevated expression of FA synthase (FAS). Inhibition of FAO by etomoxir and FAS by orlistat inhibited β-oxidation and *de novo* FA synthesis without significantly altering glucose metabolism. These effects were associated with cell cycle arrest in G0/G1 and reduced cell proliferation (43, 85). Etomoxir-mediated inhibition of FAO modestly increased the amount of lactate generated without altering glucose metabolism, to suggest that the inhibition of FAO in myeloma cells did not result in an adaptive mechanism to sustain energy homeostasis. FAS was elevated in ~70% of MM patients compared to healthy volunteers and inhibition of FAS by cerulenin promoted apoptosis (99). MMCLs and primary MM cells overexpress FAS to promote their survival and proliferation. MM patients have been reported to exhibit greater levels of saturated FAs and n-6 polyunsaturated FAs (PUFA), compared to healthy controls. *Acetyl-CoA synthetase 2 (ACSS2)* is overexpressed in MM cells derived from obese patients and contributes to myeloma progression (100). ACSS2 interacts with the oncoprotein interferon-regulated factor 4 (IRF4), and enhances IRF4 stability and IRF4-mediated gene transcription through activation of acetylation. The importance of *ACSS2* overexpression in myeloma was confirmed by finding that an ACSS2 inhibitor reduced myeloma growth *in vitro* and in a diet-induced obese mouse model. The findings demonstrated a key impact for obesity-induced ACSS2 on myeloma progression and could be important for other obesity-related malignancies. Glioma cells were incubated with tetradecylthioacetic acid (T11111141), which cannot be β-oxidized, and the oxidizable FA palmitic acid (PA), in the presence of **l**-carnitine and the carnitine palmitoyltransferase inhibitors etomoxir and aminocarnitine. **l**-carnitine partially abolished PA-mediated growth reduction of glioma cells, whereas etomoxir and aminocarnitine enhanced the anti-proliferative effect of PA (101). Similarly, Samudio et al. demonstrated that inhibition of FAO with etomoxir or ranolazine reduced the proliferation and sensitized human leukemia cells to ABT-737-induced apoptosis (102). The conventional view has been that cancer cells predominately produce ATP by glycolysis, rather than by oxidation of energy-providing substrates. Mitochondrial uncoupling, i.e., continued reduction of oxygen without ATP synthesis, may obviate the ability of oxygen to inhibit glycolysis and promote the preference for glycolysis by shifting from pyruvate oxidation to FAO.

## 4 Oncogenic *MYC* and myeloma metabolism

Transcription factors of the *MYC* family are deregulated in up to 70% of all human cancers and *MYC* deregulation is a determinant of myeloma progression (103–105). Oncogenic levels of *MYC* regulate almost every aspect of cellular metabolism. *MYC* plays a key role in the regulation of aerobic glycolysis and activates glycolytic genes not only by transcription, but also through alternative splicing. In addition, enhanced *MYC* expression upregulates the level of glutamine transporters and suppresses inhibition of glutaminolysis (77, 94, 106). Glutamine depletion led to the rapid loss of the MYC protein, independent of *MYC* transcription and post-translational modifications. However, *MYC* loss was dependent on proteasomal activity and this loss was paralleled by an equally rapid induction of apoptosis (106). *MYC* transcription is upregulated in certain MM cells, especially during later stages of disease. The estimated 24-month progression-free survival was found to be significantly shorter in patients with intermediate to high *MYC* expression compared with patients with low *MYC* expression (107). However, this did not translate into a significant difference in OS. Somewhat different results were presented by Chng et al. which indicated that patients with *MYC*-expressing tumors had a significantly shorter OS (105). Chng et al. further reported that nearly all tumors with *RAS* mutations expressed a *MYC* activation signature. *MYC* activation, assessed by gene expression signature or immunohistochemistry was associated with hyperdiploid MM, and shorter survival even in tumors non-proliferative.

## 5 Impact of the hypoxic microenvironment on metabolism in myeloma cells

MM cells are exposed to different levels of oxygen and nutrients leading to metabolically heterogeneous phenotypes that differentially respond to therapeutic intervention (108–112). Hypoxia-inducible factors (HIFs), e.g., HIF-1α, are stabilized (108, 111, 113) within the TME and HIF-1α activation intensifies conversion of pyruvate into lactate instead of the oxidation of pyruvate in mitochondria. HIF-1α is also essential in regulating vascular endothelial growth factor (VEGF) which is associated with a poor prognosis in MM (114). HIF-1α was reported to be increased in MM as compared to controls (115, 116). The expression of *HIF-1α* was also correlated with serum levels of VEGF, basic fibroblast growth factor (bFGF) and angiopoietin-2 (Ang-2) (117–125). Gene expression datasets indicated that *HIF-1α* and *HIF-2α* were enriched in cells from NDMM patients compared to those from healthy donors (45, 126, 127). IMiDs treatment has been shown to decrease *HIF-1α* expression within the BM indicating that HIF-1α could also serve as a target in MM (128).

**Figure 1:**
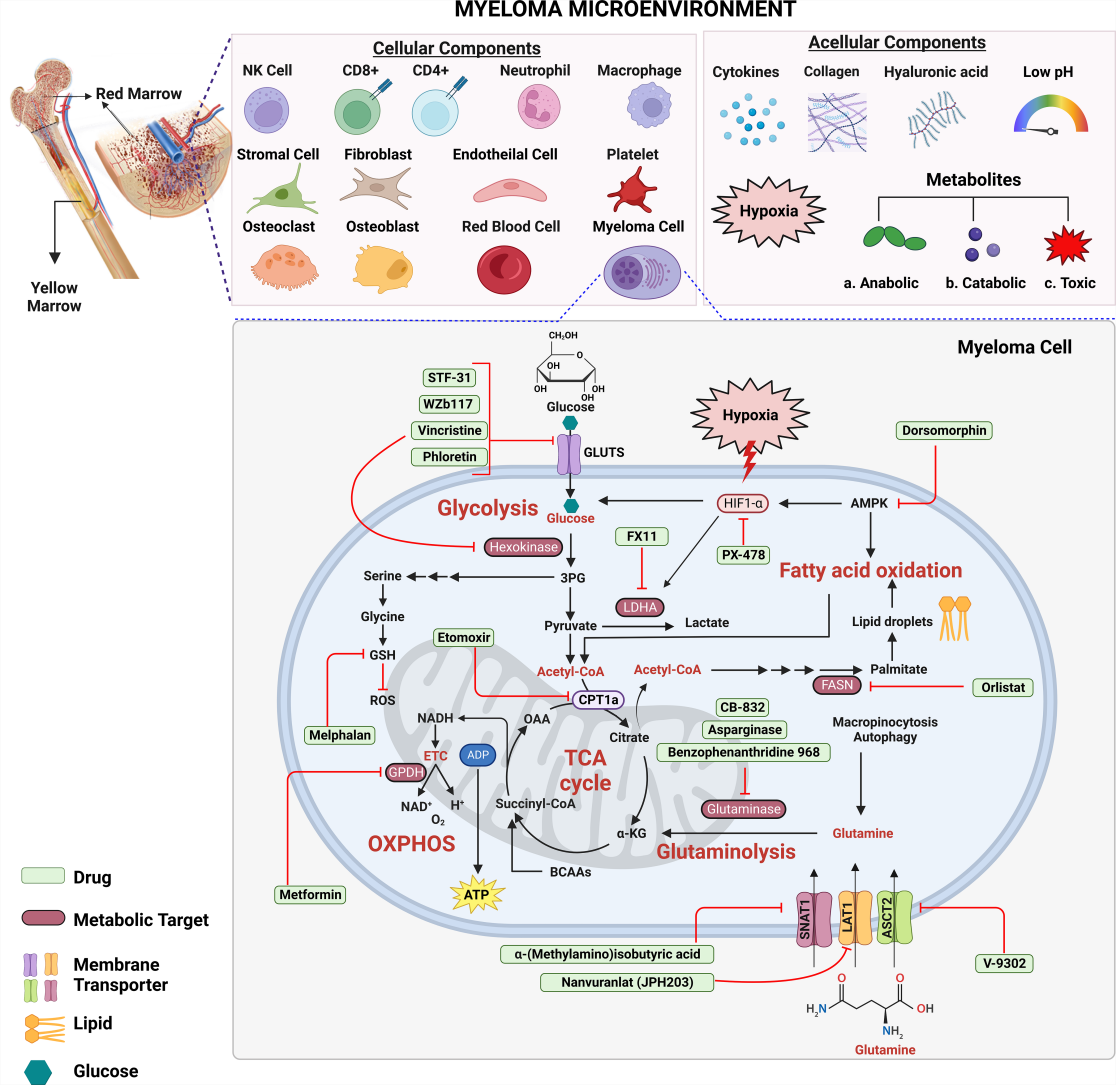
Targeting metabolic energy supply chains to enhance anti-myeloma therapy. Multiple myeloma cells use glucose as a primary source of energy followed by glutamine and fatty acids. Within cytosol, glucose is metabolized *via* glycolysis into two molecules of pyruvate and adenosine triphosphate (ATP) each. Next, pyruvate is transported across the mitochondrial matrix and is oxidized *via* TCA cycle to acetyl-CoA. Glutamine is transported across the membrane *via* transporters where it is metabolized into α-ketoglutarate (α-KG) *via* glutaminolysis. Oxidation of fatty acids results in breakdown of fatty acids into acetyl-CoA units. Which supplies energy to other tissues when glycogen stores are depleted. Each metabolic step releases energy in the form of electrons which are accepted by the electron transport chain to generate even more ATP through oxidative phosphorylation (OXPHOS). Metabolism targeting drugs (green) inhibit the key metabolic steps in glycolysis, TCA cycle, fatty acid synthesis, OXPHOS and glutaminolysis. **Figure 1** is an original image created with Biorender.com, Toronto, Canada.

The TME consumes vast amounts of oxygen that is required for aerobic glycolysis within tumor cells (129) (**Figure 1**). Hypoxia increases anaerobic glycolysis by activating HIF (130) and hypoxia-induced *LDHA* and *HKII* promote PI-resistance in MM cells (45). It was also shown that activation of miR-210 due to hypoxia significantly reduced tumor susceptibility to CD8^+^ cytotoxic T-lymphocytes (CTLs) by downregulating *PTPN1, HOXA1*, and *TP53I11* in melanoma and lung cancer cells (131). Hypoxia inducible miR-210 significantly downregulated *PTPN1* and *TP53I11* in MMCLs (132). Moreover, the HIF-inducible factor adrenomedullin is released from MM cells and stimulates vascular endothelial cells to express the angiogenic receptors CRLR and RAMP2 to promote angiogenesis (133). HIF-1α regulates interleukin (IL)-32 release from myeloma cells that is taken up by osteoclasts (134). The hypoxia-inducible p38-cyclic adenosine monophosphate (AMP) response element-binding protein (CREB)-Dickkopf-related protein 1 (DKK1) axis and upregulation of the HIF-1α-inducible MM SET domain-containing histone methyltransferase (MMSET) suppress osteoblastic bone formation (135). Taken together, hypoxic stress creates a favorable environment for myeloma survival by regulating chemotaxis, stimulating osteoclasts and endothelial cells, and inhibiting osteoblasts (**Figure 1**).

Bortezomib inhibits HIF-1α at the transcriptional level which in turn impairs recruitment of the coactivator CBP/p300 (136). The effects of PIs are attenuated within the hypoxic TME possibly due to reduced endoplasmic reticulum (ER) stress. In addition, the degradation of unfolded proteins normally mediated by proteasomes may be alternatively removed by autophagy.

HIF-inducible HKII activates autophagy by inhibiting mammalian target of rapamycin (mTOR) signaling (137). HKII is a promising therapeutic target and HKII inhibitors may increase the efficacy of anti-myeloma agents.

## 6 Metabolic alterations that alter anti-myeloma immunity

MM cells remodel the BM milieu to reshape the TME and negatively impact effectors of anti-tumor immunity (138–140). Deregulated tumor metabolism impairs the functional capacity of neighboring immune cells and compromises their differentiation (141–147). Adaptations within the TME create a competition for nutrients required by myeloma cells with their neighboring non-tumor cells. MM cells outcompete neighboring cells for nutrients to enhance tumor growth and impair anti-tumor immunity. Further dissecting the metabolic requirements of tumor and non-tumor cells in the TME may enhance immunotherapeutic responses. In addition, a disrupted vasculature deprives the TME of adequate blood supply and enhances competition between tumors and infiltrating immune cells (148). In MM, CD4^+^ and CD8^+^ T-cells form the primary immune defense, however, tumor-induced remodeling of the TME is unfavorable to T-cells due to nutrient deprivation, acidosis, and the accumulation of toxic metabolites (146) (**Figure 1**). The hypoxic microenvironment also upregulates PD-L1 expression on tumor cells through HIF-1α and a hypoxia response element (HRE) (149, 150). In MM, PD-L1 expression has been shown to be upregulated on malignant PCs (151, 152). NK cells from MM patients express PD-1, in contrast to NK cells from healthy individuals, which suppresses NK cell cytotoxicity (153). Immune cells take up and utilize amino acids, e.g., l-arginine, a non-essential amino acid present in macrophages and DCs, and lipids that are necessary for functional activity (154–157). As a product of aerobic or anaerobic glycolysis in tumors, lactic acid induces *VEGF* expression and M2-like polarization of tumor-associated macrophages (158). Tumor secretion of lactate also promotes overexpression of arginase I isoform in macrophages and is associated with immunosuppression. Lactate is not only a secondary product of cancer metabolism, but also promotes immune evasion through various mechanisms (159–161). Adenosine, and other products of cancer cell metabolism, interfere with the antitumor effect of infiltrating T-cells (162, 163). Tryptophan metabolites, especially kynurenine generated through indoleamine 2,3-dioxygenase (IDO1), have been shown to modulate T-cell activity (141–144). Kynurenine, produced by both *IDO-1* and tryptophan-2,3-dioxygenase-2 (TDO-2), upregulated the PD-1 co-inhibitory pathway on activated CD8^+^ T-cells *in vitro* compared with vehicle-treated cells (140). Since tryptophan catabolites suppress immunity, blocking tryptophan catabolism with IDO inhibitors is a potential anticancer strategy (164). Targeting the tryptophan catabolic kynurenine pathway using immune-based approaches has been shown to enhance antitumor immunity and cytotoxicity in MM (165).

## 7 Conclusions

A century after Warburg discovered that tumor cells switch from mitochondrial respiration to glycolysis to generate energy, even under aerobic conditions, cancer metabolism remains perplexing. Myeloma cells exhibit a metabolic phenotype characterized by enhanced glycolytic flux for ATP production, glucose to lactate conversion and reduced mitochondrial OXPHOS (166–168). In contrast to healthy, differentiated cells, which rely on mitochondrial OXPHOS to generate energy, cancer cells rely on aerobic glycolysis. The switch to aerobic glycolysis may represent an adaptation to facilitate the uptake of nucleotides, amino acids, and lipids required for replication (169–171). Reprogramming of the metabolic pathways that contribute to tumor growth has exposed molecular vulnerabilities and actionable targets that can be exploited (**Table 1**). Warburg described aerobic, *not anaerobic*, glycolysis and therefore there must exist factors other than HIF1-α which elicit the Warburg effect. In addition, HIF1-α is not expressed in MM cells unless grown under hypoxic conditions. Recent work by Abdollahi et al. (172, 173) demonstrated a role for PRL-3 in the induction of glucose uptake and enhanced glycolysis. Importantly, this effect was not mediated through HIF1-α, c-Myc or AMPK, but rather through STAT1 and STAT2. In hypoxia there was synergy between HIF1-α and PRL-3 in promoting glycolysis. Contrary to HIF1-α, PRL-3 does not seem to reduce OXPHOS, and recent research has shown that many hematological cancers do not downregulate OXPHOS activity (174).

Proteasomes are central to the protein degradation machinery of eukaryotes (175, 176). Healthy and transformed cells depend on proteasomes to control the level of proteins linked to metabolism, survival and proliferation (177). Based upon these findings, over the past two decades PIs have emerged as a transformative anti-myeloma therapy that has improved patient OS and quality-of-life. Proteasome abundance and catalytic activity is controlled at the level of assembly and is finely tuned by adaptations in cellular metabolism (177). Sequencing of PCs from NDMM patients has shown that MM is frequently dominated by *RAS* (43% of patients) and nuclear factor kappa B (NF-κB) pathway (17%) mutations (178). Malignant PCs undergo extensive metabolic reprogramming during myelomagenesis that is enhanced by *KRAS*, *NRAS*, and *BRAF*-activating mutations to elevate proteasomal capacity and reduce ER stress (179). *Ras* and related proteins are mutated or deregulated in many solid tumors, but PIs are ineffective against these cancers (180). Future studies are needed to decipher how solid tumors reprogram cell metabolism to evade the cytotoxic effect of PIs. Novel agents and drug delivery systems that target cancer metabolism may broaden the therapeutic impact of PIs in rationally-designed drug cocktails that improve patient survival.

## Author contributions

JD, PR, and KG developed the concept, wrote, edited, made substantial contributions, and approved the final version of the manuscript.

## Glossary

**Table:**

α-KG	α-ketoglutarate
ACSS2	Acetyl-CoA synthetase 2
AKT	A serine/threonine kinase from the ***t*** hymoma cell line AKT-8, derived from the Stock ***A*** strain ***k*** AKR mouse. Also known as Protein kinase B (PKB)
AML	Acute Myeloid Leukemia
AMP	Adenosine monophosphate
Ang2	Angiopoietin-2
ASO	Antisense oligonucleotide
ASCT2	Alanine, serine, cysteine transporter 2
ATP	Adenosine triphosphate
bFGF	Basic fibroblast growth factor
BiTE	Bispecific T cell engager
BM	Bone marrow
CAR	Chimeric antigen receptor
CD	Cluster of differentiation
CTL	Cytotoxic T-lymphocyte
CREB	Cyclic AMP response element-binding protein
D2-HG	{{sc}}d{{/sc}}-2-hydroxyglutarate
DKK1	Dickkopf-related protein 1
DLBCL	Diffuse large B-cell lymphoma
DNA	Deoxyribonucleic acid
DHA	Docosahexaenoic acid
EHA	Eicosapentaenoic acid
ER	Endoplasmic reticulum
FA	Fatty acid
FAO	Fatty acid oxidation
FAS	Fatty acid synthase
FDG	Fluorodeoxyglucose
GLUT	Glucose transporter protein type
GSH	Glutathione
HKII	Hexokinase II
HIF	Hypoxia-inducible factor
HRE	Hypoxia response element
HIV	Human immunodeficiency virus
IDO	Indoleamine 2,3-dioxygenase
IL	Interleukin
IRF4	Interferon-regulated factor 4
LAT1	L-Type Amino Acid Transporter 1
LDHA	Lactate dehydrogenase A
LSC	Leukemic stem cell
mTOR	Mammalian target of rapamycin
mTORC1	Mammalian target of rapamycin complex 1
miR	MicroRNA
M-spike	Monoclonal or Myeloma protein spike or paraprotein
MGUS	Monoclonal gammopathy of unknown significance
MM	Multiple myeloma
MMSET	MM SET domain-containing histone methyltransferase
NADPH	Nicotinamide adenine dinucleotide phosphate
NDMM	Newly diagnosed MM
NEK2	NIMA-related kinase 2
NIMA	Never in mitosis gene A
NHL	Non-Hodgkin’s lymphoma
NK	Natural killer
NF-κB	Nuclear factor kappa B
OXPHOS	Oxidative phosphorylation
PE	Phosphatidylethanolamine
PET	Positron emission tomography
coactivator-1β̣PFK	Phosphofructokinase
PKM1	Pyruvate kinase M1
PKM2	Pyruvate kinase M2
PI3K	Phosphatidylinositol 3-kinase
PI	Proteasome inhibitor
PUFA	Polyunsaturated fatty acid
R-CHOP	Rituximab, cyclophosphamide, hydroxydaunorubicin HCl, vincristine (Oncovin) and prednisone used to treat both indolent and aggressive forms of NHL
ROS	Reactive oxygen species
RRMM	Relapsed and/or refractory MM
siRNA	Small interfering RNA
SLC	Solute carrier
STAT-3	Signal transducer and activator of transcription 3
SGLT	Sodium-dependent glucose transport
TCA	Tricarboxylic acid
TCR	T-cell receptors
TDO-2	tryptophan-2,3-dioxygenase-2
TP53	Tumor protein 53
UTR	Untranslated region
VEGF	Vascular endothelial growth factor
WT	Wildtype.
